# Parameters for the Analysis of Social Bonds in Horses

**DOI:** 10.3390/ani8110191

**Published:** 2018-10-27

**Authors:** Riccarda Wolter, Volker Stefanski, Konstanze Krueger

**Affiliations:** 1 Department of Zoology, University of Regensburg, Universitätsstraße 31, 93053 Regensburg, Germany; Riccarda.Wolter@gmx.de; 2 Department Equine Management, Faculty Agriculture, Economics and Management, Nürtingen-Geislingen University, Neckarsteige 6-10, 72622 Nuertingen, Germany; 3 Institute of Animal Husbandry and Animal Breeding, University of Hohenheim, Garbenstr. 17, 70599 Stuttgart, Germany; volker.stefanski@uni-hohenheim.de

**Keywords:** feral horses, mutual grooming, social bonds, social bond analysis, spatial proximity

## Abstract

**Simple Summary:**

As more and more horses are being kept in group housing, it is important to take the horses’ likes and dislikes of other group members (their social bonds) into account to ensure the animals’ well-being and minimise aggressive encounters between group members. Methods for the analysis of social bonds need to be improved and integrated into horse welfare protocols. We observed social behaviour and spatial proximity in 145 feral horses, comprising five groups of Przewalski’s horses and six groups of feral horses. We found that 15 h of observation per group provided robust and reliable data for the analysis of social bonds. Either a combination of counts of friendly approaches and mutual grooming between pairs of horses, or the analysis of the horses’ nearest neighbors through measurements of the animals spatial proximity, are suitable ways of gaining insight into the horses’ social relationships.

**Abstract:**

Social bond analysis is of major importance for the evaluation of social relationships in group housed horses. However, in equine behaviour literature, studies on social bond analysis are inconsistent. Mutual grooming (horses standing side by side and gently nipping, nuzzling, or rubbing each other), affiliative approaches (horses approaching each other and staying within one body length), and measurements of spatial proximity (horses standing with body contact or within two horse-lengths) are commonly used. In the present study, we assessed which of the three parameters is most suitable for social bond analysis in horses, and whether social bonds are affected by individual and group factors. We observed social behaviour and spatial proximity in 145 feral horses, five groups of Przewalski’s horses (*N* = 36), and six groups of feral horses (*N* = 109) for 15 h per group, on three days within one week. We found grooming, friendly approaches, and spatial proximity to be robust parameters, as their correlation was affected only by the animals’ sex (GLMM: *N* = 145, *SE* = 0.001, *t* = −2.7, *p* = 0.008) and the group size (GLMM: *N* = 145, *SE* < 0.001, *t* = 4.255, *p* < 0.001), but not by the horse breed, the aggression ratio, the social rank, the group, the group composition, and the individuals themselves. Our results show a trend for a correspondence between all three parameters (GLMM: *N* = 145, *SE* = 0.004, *t* = 1.95, *p* = 0.053), a strong correspondence between mutual grooming and friendly approaches (GLMM: *N* = 145, *SE* = 0.021, *t* = 3.922, *p* < 0.001), and a weak correspondence between mutual grooming and spatial proximity (GLMM: *N* = 145, *SE* = 0.04, *t* = 1.15, *p* = 0.25). We therefore suggest either using a combination of the proactive behaviour counts mutual grooming and friendly approaches, or using measurements of close spatial proximity, for the analysis of social bonds in horses within a limited time frame.

## 1. Introduction

Keeping horses in social groups has become increasingly popular and several welfare assessment protocols have been created to monitor the well-being and the behaviour of the group members [1,2,3,4]. When group compositions are managed to ensure the welfare of the group members, it is important to consider social bonds [5]. As in many mammals [6,7], horses form long lasting social relationships with group members, so-called social bonds [8,9,10]. Horses prefer to affiliate with a small subset of the available group members [8,9,11]. The best method of measuring social bonds in horses is still not clearly defined and it can be performed in many different ways. The measurement of the type, frequency, and duration of friendly (affiliative) social behaviour can be useful. In many species, it is common practice to use the frequency of mutual grooming between particular individuals [7,12,13,14]. However, because mutual grooming behaviour is rare in horses and therefore does not allow for a robust analysis of social bonds, some studies consider other measures, such as friendly approaches [5,11] or spatial proximity [3,10,15].

The feral horse appears to be a good model species for the discussion of the evaluation of social bonds. The feral horses’ group formation is little affected by human management, they form stable social bonds [16,17,18,19] and their mutual grooming frequencies are low and show individual and seasonal variation. In mutual grooming, one horse approaches another and begins sniffing or nuzzling the approached horse along the dorsal surface of the body from the neck, over the withers and the back, to the rump [20]. Some feral horses groom mutually at least once an hour, while others in the group may only participate once every ten hours, and some do not groom at all [20]. For all horses, the frequency of grooming peaks in the spring when winter coats are shed [20] and participants receive coat care in areas of the body that are difficult to reach by self-grooming [21,22].

Datasets for the analysis of social bonds were supplemented by measurements of spatial proximity, for example when horses are grazing and resting [9,12,13,15,23], or spatial proximity was used exclusively [3,10]. Authors differ in their methods for measuring spatial proximity regarding the distance between two individuals and the timing of observation intervals [5,8,10,24,25]. To sum up, the distance of two animals standing within two body lengths of each other appears to be used most often [5,8,25] and the sampling time interval of ten min appears to guarantee the independence of samples when calculating spatial proximity, as horses show a mean latency of changing the spatial distribution of group members every 8 min [25].

Besides spatial proximity, mutual affiliative approaches were an additional parameter for measuring social bonds [5,11]. Mutual approaches were categorized as affiliative or “friendly” and were considered to be indicative of the desire for friendly interaction and proximity to other animals [5,7] when a receiver reciprocated an approach or behaved neutrally, i.e., not challenging or fleeing from the approaching animal [11].

The aim of this study was to explore the most reliable parameter, or combination of parameters, for the analysis of social bonds in a species that seldom grooms, such as the horse. We compared the frequencies of mutual grooming, friendly approaches, and close proximity, in pairs of horses. We collected this data by observing five groups of Przewalski’s horses and six groups of feral horses, all living under semi-wild conditions. Przewalski’s horses and domestic horses (*Equus caballus*) are considered to show similar social behaviour [25] and Przewalski’s horses, most likely, descended from domestic horses 5000–6000 years ago [26]. Nevertheless, we tested whether the frequencies of social behaviour and spatial proximities are similar in the Przewalski’s horses and the feral horses, as evolutionary, genetic, and environmental differences could have influenced the behaviour of the test horses of the present study.

Therefore, we addressed the following questions: (1) Are potential correlations between the individual animals’ frequencies of grooming, friendly approach, and close proximity with group members affected by the horses’ breeds (Przewalski’s horse or feral horse [26]), their aggressiveness [27], their social rank [15,19], their sexes, the groups, the group sizes, the group composition (harem, mare group, bachelor group), or by the individuals themselves [9,28,29,30,31,32]? (2) Are there correlations between the frequency of mutual grooming, the frequency of friendly approaches, and the frequency of staying in close proximity in pairs of horses [5,7,8,11,24,25]?

## 2. Methods

### 2.1. Animals

We observed 145 feral horses (Table 1), five groups of Przewalski’s horses (*Equus ferus przewalskii, N* = 36) and six groups of feral horses (*Equus ferus caballus*, *N* = 109). All groups were stable for at least 12 months. The Przewalski’s horse groups were kept in semi-reserves in Germany, in the Stadtwald Augsburg, in Campo Pond Hanau, in Hohe Warte Gießen, and in Ludwigsthal Bavarian forest. The first group (group P-1, *N* = 5) was a bachelor group consisting of male uncastrated animals (i.e., stallions), the second group (group P-2, *N* = 7) was a mare group consisting of females, the third group was a harem group (group P-3, *N* = 6) with one stallion, four mares, and one yearling, the fourth group (group P-4, *N* = 9) was a mare group, and the fifth group (group P-5, *N* = 9) was a harem composed of one stallion, six mares, and two yearlings. In addition, we observed the behaviour of 109 feral horses, living in six different harem groups (group F-6: *N* = 23, group F-7: *N* = 10, group F-8: *N* = 12, group F-9: *N* = 19, group F-10: *N* = 26, group F-11: *N* = 19). These were among about 300 feral horses known as ‘Cavalli di Esperia’. The observed population roams freely in the Abruzzi Mountains near Frosinone in Italy. The horses’ ages ranged between 1 and 28 years, according to previous studies [11,33], but precise ages were only known for half of the animals. All groups were composed of one stallion, several females, and their male and female offspring and changed in group composition for about 15% each year.

Freeze brands (i.e., white numbers, dorsal at the animals’ torso), hot brands, colourations, and body proportions were used to identify the individual horses. The Przewalski’s horses were well known to the park rangers, and were registered at the European Conservation Breeding Program (Europäisches Erhaltungszuchtprogramm, EEP). Their social histories were comparable: they were all born in small harem groups in zoo housing. The feral horses were observed annually for previous studies and the majority of the animals had been individually known to the research group since 2008. All of the horses fed on the natural vegetation in the areas, and received additional hay during winter when the natural food supply was insufficient. Foals were excluded from the evaluations because of the special status of foals in social horse groups [34].

### 2.2. Data Sampling

Data (Table S1) were collected from September 2012 until August 2013 for group P-1 and P-2, and in April and May 2015 for the groups P-3, P-4, and P-5. The feral horses in Italy, groups F-6, F-7, F-8, and F-9, were observed in May 2010, and the groups F-10 and F11 in June 2014. Fifteen hours of continuous observations per group were distributed evenly over the daylight hours of at least three days within one week.

The observers stayed about 20–200 m away from the animals, depending on the horses’ spatial distribution. Binoculars were used to support identification, if needed. As the horses were accustomed to people, their behaviour was not influenced by the observers’ presence.

The comparatively small Przewalski’s horse groups were observed by one person, who recorded the behaviour on paper. Group P-5 was observed by two persons, with one person observing the group at a time.

The large feral horse groups were observed by eight persons in total. Four observed one group at a time and split into pairs. The two observers cooperated in collecting information and animal identities, and while A was writing, B continued observing. After the observation, an inter-observer reliability test was used to compare the two observer pairs for each feral horse group. The median Spearman correlation coefficient for the inter observer reliability in the six groups was *r_s_* = 0.89 (*Min* = 0.76, *Max* = 0.98).

Affiliative and agonistic behaviour (for further definition see below) was collected by continuous recording ad libitum [35]. Additionally, the spatial proximity of the animals (see below) was documented by scan sampling [35] every ten min. We recorded the frequency of grooming behaviour and staying in close proximity to group members that each individual animal showed within its group. Furthermore, we evaluated how often affiliative behaviour was displayed and whether particular pairs of animals stayed in close proximity to each other within each group.

### 2.3. Affiliative Behaviour

#### 2.3.1. Mutual Grooming

**Figure 1:**
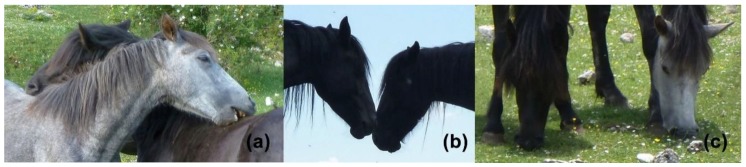
Pairwise involvement in (**a**) mutual grooming, (**b**) friendly approach, and (**c**) spatial proximity.

Mutual grooming was defined as two horses standing beside each other, usually head-to-shoulder or head-to-tail, grooming each other’s neck, mane, rump, or tail by gentle nipping, nuzzling, or rubbing [36] (Figure 1a).

#### 2.3.2. Affiliative Approaches

Affiliative approaches (Figure 1b) were counted when a horse approached and stayed within one body length of another horse, when an approach elicited a reciprocal affiliative reaction (the approached animal moving towards the approacher) or a neutral reaction of the approached horse (the approached animal did not move). Approaches that resulted in grooming were not considered, so that the friendly approach and grooming data were mutually exclusive.

### 2.4. Agonistic Behaviour

We counted agonistic behaviours of the horses, such as threats to bite or to kick, bites, kicks, chases, retreats, and approaches that elicited a retreat of the approached horse [36,37]. The mean aggression rate per group per horse in 15 h of observation is given in Table 1. We divided the total numbers of aggressive behaviour displayed within 15 h in a particular group by the number of members in the group.

### 2.5. Social Rank

The horses’ social ranks were analysed, as described by Krueger et al. [33]. We calculated the social rank of each animal from their agonistic encounters using an average dominance index (ADI), as follows. The dominance index per pair of individuals, *w_ij_* is the frequency an individual won against a certain opponent divided by the frequency of agonistic encounters between the pair, thus *w_ij_* = *x_ij_*/(*x_ij_* + *x_ji_*). Wins were counted for the initiator of an encounter when an approached or challenged animal retreated for one step or more. We excluded a pair from the analysis if the two individuals were not involved in an encounter. The average dominance index of an individual is the average of all its dominance indices with all its interaction partners, thus 1/*N* Σ*_j_ w_ij_*. The ADI can range between 0 and 1. Therefore, a higher value indicates a higher rank in the hierarchy [38].

Not only the type of agonistic behaviour, but also the reaction of the receiver is decisive in counting wins and losses. For example, an animal may respond by retreating both when it is being kicked and when it is approached. In both cases, the receiver loses and the initiator wins. This method enables all agonistic behaviour types to be used, irrespective of their frequency, and provides the largest possible sample size for the rank evaluation [38]. We chose the ADI for its reliability and computational simplicity. Simulations showed that the ADI can deal with missing data between pairs of animals and still provides more reliable results then comparable dominance assessment methods [38].

### 2.6. Spatial Proximity

Horses standing within two distance categories were considered when evaluating close spatial proximity: (I) body contact and (II) being within two horse-lengths (Figure 1c). A graph of the position of all group members was drawn every ten min during observation periods to determine the proximity of the individuals [33]. This interval was chosen according to the study by Feh [39], which shows that, in grazing horses, the probability of having the same individual in close proximity drops significantly after 8 min.

### 2.7. Statistical Analysis

The statistical analysis and the figures were done with the R-Project statistical environment (R Development Core Team 2018), packages “R commander” and “glm(stats)”. The data are provided as Supplementary Material (Table S1). The tables were drawn with Excel 2007. Some of the data were not normally distributed (Shapiro-Wilk Test). Therefore, we applied non-parametric tests. We applied a Generalized Linear Mixed Model (GLMM) to compare the fixed effects of mutual grooming, friendly approaches, and spatial proximity, and the random effects of animals ID, sex, rank, aggressive behaviour, groups, group composition, and group size. We applied the following formula = Grooming ~ Approach × Proximity/Przewalski − Feral Horse/Aggression/Rank/Sex/Group/Group Size/Group Composition/Individual, family = gaussian. The model with the best fit (with the lowest AIC) was chosen after stepwise removal of factors. An additional GLM was calculated to analyze the effect of the group size on the frequency of grooming, friendly approaches, and spatial proximity. Complete and reduced models are listed in the Supplementary Material (File S1). All tests were two-tailed and the significance level was set at 0.05.

### 2.8. Research Ethics

All of the horse owners offered their horses and their participation in the non-invasive observations of their free will. They were informed about the test procedure and the intended publication of the data before the observations and agreed with both the procedure and the publication of the data. The non-invasive observations did not cause the horses any pain, suffering, or damage and were in agreement with the German and the Italian animal welfare regulations. We obtained oral consent from the animal welfare boards of the test regions that no permits were needed for the study observations.

## 3. Results

### 3.1. Effects on the Correlation between Grooming, Friendly Approaches and Close Proximity of Individual Animals

The correlation between the animals’ frequency of grooming, friendly approaches, and staying in close proximity was affected by the animals’ sex (GLMM: *N* = 145, *t* = −2.7, *p* = 0.008), with stallions showing a higher number of mutual grooming and friendly approaches than mares (mutual grooming: stallions *N* = 15, *median* = 10.75, *min* = 3, *max* = 33, mares *N* = 130, *median* = 4, *min* = 0, *max* = 29; friendly approaches: stallions *N* = 15, *median* = 16, *min* = 4, *max* = 40; mares *N* = 130, *median* = 5, *min* = 0, *max* = 35). Furthermore, the correlation between grooming, friendly approaches, and spatial proximity was affected by the group size (GLMM: *N* = 145, *t* = 4.26, *p* < 0.001). The smaller the group size the more approaches were shown per animal within the group (GLM: *N* = 145, *t* = −4.49, *p* < 0.001), and the more each of the horses stayed in close proximity with other group members (GLM: *N* = 145, *t* = −5.83, *p* < 0.001). In contrast, the larger the group was, the more the horses were observed grooming (GLM: *N* = 145, *t* = 2.108, *p* = 0.04).

Further factors, such as the horse breed (Przewalski’s horse or feral horse), the aggression rate, the social rank, the group, the group composition (harem, mare group, bachelor group, Table 1), and the individuals themselves did not have any effect on the correlation between the grooming and friendly approach behaviour and their spatial proximity.

### 3.2. Correlations between Grooming, Approach and Close Proximity in Pairs of Animals

**Figure 2:**
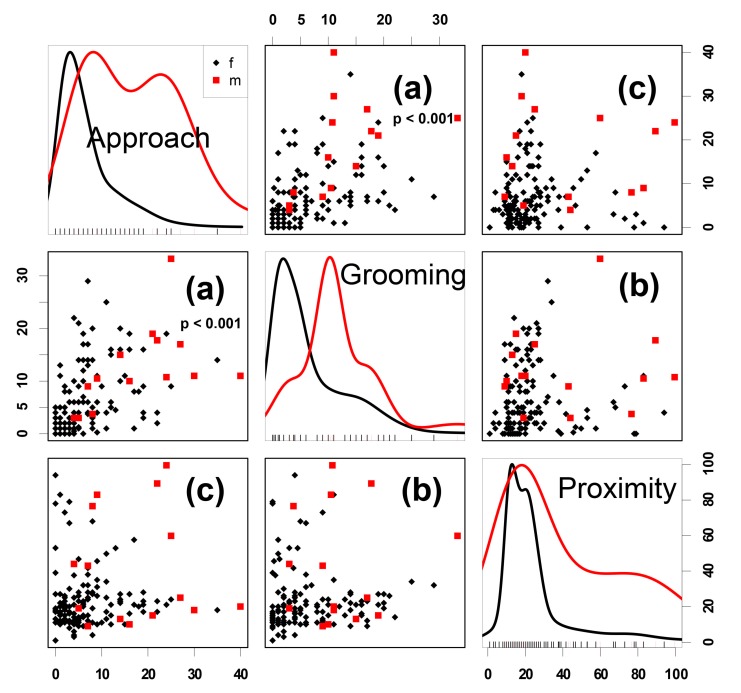
Correlations between the frequencies of grooming, friendly approaches, and being in spatial proximity of pairs of horses in each group. On the diagonal frequencies of the behaviours approaches, grooming and proximity are shown for male (red) and female horses (black). The scatterplots depict the correlations between the frequencies of pairs of parameters. The y and x axes quantify the frequencies of the parameters in the respective column (x-axis) or row (y-axis). The frequency of pairwise (**a**) grooming and friendly approaches correlate strongly, i.e., when the frequency of grooming increased, friendly approaches also increased. (**b**) grooming and proximity and (**c**) approach and proximity did not correlate, i.e., when animals stayed in close proximity some showed a low and others a high grooming and friendly approach frequency. Significant correlations are given with *p* < 0.001.

Our results show a strong correlation between the frequency of pairwise mutual grooming and friendly approaches (GLMM: *N* = 145, *SE* = 0.021, *t* = 3.922, *p* < 0.001, Figure 2a), no correlation between the animals’ pairwise mutual grooming and their close spatial proximity (GLMM: *N* = 145, *SE* = 0.04, *t* = 1.15, *p* = 0.25, Figure 2b), and a trend for a correlation between all three counts: mutual grooming, friendly approaches, and close spatial proximity (GLMM: *N* = 145, *SE* = 0.004, *t* = 1.95, *p* = 0.053).

## 4. Discussion

In pairs of horses, mutual grooming exchanges correlated with the exchange of friendly approaches, but not with being in close proximity with particular group members.

### 4.1. The Value of Grooming, Affiliative Approaches and Spatial Proximity for the Measurement of Social Bonds in Horses

Social bonds have been investigated in primate societies [7,14,12,40,41], dolphins [42], rodents [43], birds [44], and horses [5,8,9,10,11]. The formation of social bonds in general has been considered to promote fitness, e.g., it can increase the bonded animals’ reproductive success (feral horses [5]; Macaques [45]) and their offspring survival (Baboons [7]; feral horses [5]). The meaning of the parameters mutual grooming, friendly approaches, and close proximity to group members in the analysis of social bonds in horses is under debate.

Similar to social licking in cows [46,47,48], mutual grooming between horses has been claimed to promote social bonding [5,8,9,11], to be an appeasement behaviour [22,49,50], and to reduce aggression between group members [27,51,52,53]. Grooming has been claimed to be reciprocated in breeding partners and may also serve partner control and partner choice [14]. Grooming frequencies may be affected by the parasite load and the season. The horses may groom more frequently when the winter fur is shed or when parasites cause skin damage [21,37].

Friendly approaches may promote the formation and stability of social bonds [27,51,52], and horses may exchange individual information about bonded and potentially bonded group members after approaching each other [54]. Furthermore, friendly approaches may be used to protect social bonds, as Schneider and Krueger [11] showed that high ranking female horses approach socially bonded group members and intervened in grooming encounters with other group members.

Also, spacing behaviour demonstrates social bonding, as horses that stay close to each other are more likely to prefer each other [3,10,25]. Using spatial proximity may be the only way to measure social bonds when horses cannot be identified individually, but are tracked via GPS. However, spatial proximity may be influenced by environmental factors, such as temperature [28] and insect pressure [55], and by aggressive animals avoiding some and being avoided by others [53].

Mutual grooming, friendly approaches, and spatial proximity appeared to be robust affiliative social behaviour parameters in horses, as their frequencies were affected only by the animals’ sex [9,53]. Male horses may be more strongly connected with their group members than females as they display more social behaviour, as in primates [56,57]. Furthermore, social relationships appeared to be stronger in smaller rather than in larger groups as has been claimed for social groups in general [58]. In the present study, this is demonstrated by the high frequency of friendly approaches and the horses’ staying in close proximity in small groups. The fact that grooming was more frequent in larger, potentially instable groups [58] may indicate the appeasing, aggression regulating, prosocial value of mutual grooming.

Our findings support previous studies on grooming in primates [12] and social licking in cows [46,47,48], which did not find any evidence that social rank affects the frequency of staying in close proximity to group members [31,33,59] or the frequency of friendly approaches and mutual grooming.

For a deeper understanding of the value of the parameters mutual grooming, friendly approaches, and spatial proximity in the analysis of social bonds in horses, a knock out study [56,57] is needed in which directed data of the parameters are compared with physiological stress measurements, as has been done, for example, for social licking in cows [46,47,48]. Moreover, evaluations of the effect of foals on the social bonding between group members are needed because foals are considered to have a special status within the group [34] and may be protected by several mares [5]. Furthermore, the effect of environmental factors, such as the season, food availability, and parasite load need to be considered, but were not the focus of this study.

### 4.2. The Value of Social Bond Analysis for the Management of Horse Groups

Welfare assessment protocols are created to measure the wellbeing of individual horses that are kept in group housing and include behaviour assessments [1,2,3,4]. However, social bonds are often not considered because of observation time restrictions for the protocols. For the present study, we collected the necessary behaviour data for social bond analyses in well-identified horses within 15 h. It may be necessary to reduce the observation duration further for application in animal welfare protocols. A follow up study is needed to investigate whether, in addition to friendly approaches, further proactive behaviour, such as grazing and resting together, could be combined with the mutual grooming data to analyze social bonds in horses even more quickly and precisely. Further individual factors (e.g., reproductive status and age [9]), which were missing for most of the observed horses in the present study, may also be considered. Other behaviors not found to be integral to this short term study may also be important when assessing social bonds or the development of social bonds in social species, but may require longer observation periods.

## 5. Conclusions

Observation of proactive behaviour, such as mutual grooming and friendly approaches, or of spatial proximity between group members, are suitable for social bond analysis in horses within a short time frame of 15 h. We expect a combination of friendly approaches and mutual grooming, or the measurement of spatial proximity, to be robust for the majority of horse groups, as this was the case for all the groups in the present study, even though the groups varied in their composition and the parameters of the individual animals.

## Figures and Tables

**Table 1:** Overview on the composition of the horse groups.

Name of the Group	Group Size	Horse Breed	Sex, Number of Males Females		Group Type	Average Age [Years]	Nr. Aggressive Behaviour in 15 h/Group/Horse
P-1	5	Przewalski’s	5	0	Bachelor group	2.6	2
P-2	7	Przewalski’s	0	7	Mare group	8.7	5
P-3	6	Przewalski’s	2	4	Harem	8.5	2
P-4	9	Przewalski’s	0	9	Mare Group	6.2	11
P-5	9	Przewalski’s	2	7	Harem	10.4	40
F-6	23	Feral horses	3	20	Harem	N.A.	24
F-7	10	Feral horses	3	7	Harem	N.A.	25
F-8	12	Feral horses	3	9	Harem	N.A.	25
F-9	19	Feral horses	3	16	Harem	N.A.	21
F-10	26	Feral horses	7	19	Harem	N.A.	18
F-11	19	Feral horses	4	15	Harem	N.A.	20

## Supplementary Materials

The following are available online at http://www.mdpi.com/2076-2615/8/11/191/s1, Table S1: Data for Social Bond Analysis. The table provides the complete data used for the analysis of the present manuscript; File S1: Statistical Data. The file provides the complete and the reduced Statistical Models calculated for the present study.
