# Supplementary material for: Parameters for the Analysis of Social Bonds in Horses
Source: Animals (Basel). 2018 Oct 27;8(11):191. doi: 10.3390/ani8110191 (PMC6262610; doi:10.3390/ani8110191)
Supplement: Supplementary file 1 [file animals-08-00191-s001.zip › Wolter_Supplementary_Material_1_raw_data.pdf]

Pferd	Individual	Group	Group composition	P/F	Rank	Proximity	Grooming	Approaches	Aggression	Group Size	Sex	sex nom
Marlon	1	1	1	1	1	60	33	25	5	5	1	m
Solongo	2	1	1	1	2	100	11	24	2	5	1	m
Vinter	3	1	1	1	3	89	18	22	1	5	1	m
Xaran	4	1	1	1	4	77	4	8	1	5	1	m
Kalmoek	5	1	1	1	5	83	11	9	1	5	1	m
Barioja	6	2	2	1	1	47	1	5	4	7	2	f
Abadia	7	2	2	1	5	50	1	11	2	7	2	f
Ajala	8	2	2	1	2	58	1	17	7	7	2	f
Ginger	9	2	2	1	3	42	4	7	4	7	2	f
Galinka	10	2	2	1	6	53	5	13	8	7	2	f
Sira	11	2	2	1	4	46	0	9	5	7	2	f
Ilonka	12	2	2	1	7	38	1	4	3	7	2	f
Barioja	6	3	3	1	1	11	0	4	3	6	2	f
Galinka	10	3	3	1	5	13	1	0	0	6	2	f
Ginger	9	3	3	1	2	25	2	2	5	6	2	f
Ilonka	1	3	3	1	3	22	3	1	3	6	2	f
Fury	17	3	3	1	4	19	3	5	1	6	1	m
Oleg	18	3	3	1	6	44	3	4	2	6	1	m
Maul	19	4	2	1	1	38	6	7	7	9	2	f
Chrissi	20	4	2	1	8	67	6	3	6	9	2	f
Marie	21	4	2	1	7	83	11	1	22	9	2	f
Ajala	8	4	2	1	3	68	5	8	22	9	2	f
Nele	23	4	2	1	4	94	4	0	3	9	2	f
Jana	24	4	2	1	5	78	0	0	3	9	2	f
Schweif	25	4	2	1	9	73	9	2	0	9	2	f
Sira	11	4	2	1	2	79	0	3	36	9	2	f
Abadia	7	4	2	1	6	39	0	0	2	9	2	f
Aytan	28	5	3	1	3	20	11	40	50	9	1	m
Calgary	29	5	3	1	6	22	20	14	31	9	2	f
Leila	30	5	3	1	4	10	5	12	37	9	2	f
Nadja	31	5	3	1	9	21	19	24	25	9	2	f
Fumir	32	5	3	1	1	10	6	4	47	9	2	f

Holly	33	5	3	1	2	21	15	12	44	9	2	f
Leika	34	5	3	1	5	17	9	19	51	9	2	f
Olga	35	5	3	1	7	20	8	18	50	9	2	f
Otto	36	5	3	1	8	18	11	30	23	9	1	m
1	37	6	3	2	3	30	2	3	47	23	2	f
2	38	6	3	2	14	28	1	5	19	23	2	f
3	39	6	3	2	15	25	4	7	15	23	2	f
6	40	6	3	2	17	18	1	0	16	23	2	f
7	41	6	3	2	6	27	5	0	39	23	2	f
9	42	6	3	2	11	23	4	2	7	23	2	f
10	43	6	3	2	20	25	4	11	4	23	2	f
11	44	6	3	2	7	53	3	0	48	23	2	f
12	45	6	3	2	13	43	9	7	28	23	1	m
13	46	6	3	2	12	31	6	9	15	23	2	f
14	47	6	3	2	5	34	25	11	44	23	2	f
15	48	6	3	2	10	27	20	14	16	23	2	f
16	49	6	3	2	1	35	11	9	50	23	2	f
17	50	6	3	2	2	21	6	12	20	23	2	f
18	51	6	3	2	4	25	2	4	73	23	2	f
19	52	6	3	2	19	16	0	3	12	23	2	f
20	53	6	3	2	18	20	1	2	9	23	2	f
21	54	6	3	2	16	20	0	3	30	23	2	f
22	55	6	3	2	9	27	5	1	19	23	2	f
23	56	6	3	2	8	26	2	2	34	23	2	f
24	57	6	3	2	23	32	7	6	1	23	2	f
25	58	6	3	2	21	32	29	7	2	23	2	f
26	59	6	3	2	22	26	21	5	7	23	2	f
1	60	7	3	2	2	10	10	16	44	10	1	m
2	61	7	3	2	8	11	9	10	9	10	2	f
3	62	7	3	2	3	10	1	4	26	10	2	f
4	63	7	3	2	4	8	0	3	19	10	2	f
5	64	7	3	2	5	10	11	15	42	10	2	f
6	65	7	3	2	9	13	4	11	7	10	2	f

7	66	7	3	2	10	14	4	4	2	10	2	f
8	67	7	3	2	7	4	1	8	18	10	2	f
9	68	7	3	2	6	11	6	3	17	10	2	f
10	69	7	3	2	1	4	4	4	61	10	2	f
1	70	8	3	2	2	13	15	14	27	12	1	m
2	71	8	3	2	3	23	3	19	34	12	2	f
5	72	8	3	2	7	21	8	17	30	12	2	f
6	73	8	3	2	9	12	2	4	17	12	2	f
9	74	8	3	2	1	18	14	35	24	12	2	f
10	75	8	3	2	4	14	0	6	24	12	2	f
12	76	8	3	2	8	12	17	7	17	12	2	f
13	77	8	3	2	6	10	2	1	14	12	2	f
14	78	8	3	2	10	17	2	22	23	12	2	f
15	79	8	3	2	11	15	19	6	32	12	2	f
16	80	8	3	2	5	23	4	22	41	12	2	f
17	81	8	3	2	12	26	4	9	21	12	2	f
1	82	9	3	2	6	25	17	27	17	19	1	m
2	83	9	3	2	10	14	6	5	24	19	2	f
3	84	9	3	2	16	17	8	3	13	19	2	f
4	85	9	3	2	15	14	2	4	13	19	2	f
5	86	9	3	2	14	12	1	5	22	19	2	f
6	87	9	3	2	11	21	16	19	20	19	2	f
9	88	9	3	2	9	28	1	8	25	19	2	f
10	89	9	3	2	12	23	16	14	25	19	2	f
11	90	9	3	2	19	15	0	2	9	19	2	f
12	91	9	3	2	1	24	13	7	21	19	2	f
14	92	9	3	2	5	27	19	16	28	19	2	f
15	93	9	3	2	13	11	1	3	7	19	2	f
16	94	9	3	2	2	22	14	6	27	19	2	f
17	95	9	3	2	8	16	4	4	32	19	2	f
18	96	9	3	2	7	13	0	2	17	19	2	f
19	97	9	3	2	3	24	10	14	29	19	2	f
20	98	9	3	2	4	21	2	2	21	19	2	f

21	99	9	3	2	17	19	5	2	26	19	2	f
22	100	9	3	2	18	9	0	6	20	19	2	f
1	101	10	3	2	2	9	9	7	37	26	1	m
2	102	10	3	2	17	19	4	0	23	26	2	f
3	103	10	3	2	8	17	1	1	19	26	2	f
4	104	10	3	2	9	12	0	1	11	26	2	f
5	105	10	3	2	5	6	2	4	27	26	2	f
6	106	10	3	2	3	20	2	1	16	26	2	f
7	107	10	3	2	25	12	0	2	10	26	2	f
8	108	10	3	2	1	15	10	6	21	26	2	f
10	109	10	3	2	13	12	2	3	14	26	2	f
11	110	10	3	2	6	11	2	0	10	26	2	f
13	111	10	3	2	18	11	2	8	25	26	2	f
14	112	10	3	2	21	13	0	2	15	26	2	f
16	113	10	3	2	19	17	0	0	1	26	2	f
17	114	10	3	2	14	11	8	2	13	26	2	f
18	115	10	3	2	12	10	6	9	24	26	2	f
19	116	10	3	2	16	19	3	8	19	26	2	f
20	117	10	3	2	10	15	13	7	14	26	2	f
21	118	10	3	2	4	3	2	4	32	26	2	f
22	119	10	3	2	7	9	14	5	8	26	2	f
23	120	10	3	2	11	11	1	1	19	26	2	f
26	121	10	3	2	20	11	3	3	11	26	2	f
28	122	10	3	2	23	1	0	0	9	26	2	f
32	123	10	3	2	15	21	6	6	19	26	2	f
33	124	10	3	2	22	28	14	7	36	26	2	f
34	125	10	3	2	26	18	5	2	13	26	2	f
35	126	10	3	2	24	19	5	2	11	26	2	f
1	127	11	3	2	3	15	19	21	20	19	1	m
6	128	11	3	2	8	13	9	5	5	19	2	f
7	129	11	3	2	5	19	20	8	17	19	2	f
8	130	11	3	2	6	11	4	5	14	19	2	f
10	131	11	3	2	4	9	17	7	29	19	2	f

11	132	11	3	2	9	21	10	4	38	19	2	f
13	133	11	3	2	10	16	5	1	15	19	2	f
15	134	11	3	2	11	12	9	5	6	19	2	f
17	135	11	3	2	13	15	13	1	6	19	2	f
18	136	11	3	2	7	14	22	4	13	19	2	f
19	137	11	3	2	1	16	16	16	20	19	2	f
20	138	11	3	2	2	15	16	12	25	19	2	f
22	139	11	3	2	14	23	6	5	12	19	2	f
23	140	11	3	2	16	28	4	19	36	19	2	f
24	141	11	3	2	17	17	4	11	18	19	2	f
25	142	11	3	2	15	24	9	25	46	19	2	f
26	143	11	3	2	12	27	14	8	37	19	2	f
27	144	11	3	2	18	23	17	6	10	19	2	f
28	145	11	3	2	19	20	12	6	8	19	2	f
