# Supplementary material for: Parameters for the Analysis of Social Bonds in Horses
Source: Animals (Basel). 2018 Oct 27;8(11):191. doi: 10.3390/ani8110191 (PMC6262610; doi:10.3390/ani8110191)
Supplement: Supplementary file 1 [file animals-08-00191-s001.zip › Wolter_Supplementary_Material_2_Statistical_Models.pdf]

Supplementary Material 2

Statistical Models

Parameters for the analysis of social bonds in horses

Wolter R*. Stefanski V., Krueger K.

* Corresponding author: Riccarda.Wolter@gmx.de, Telephone: +49 6181 – 440 8789

```
Complete GLMMCall:


glm(formula = Grooming frequency ~ Approach frequency * Proximity frequency/Aggression/Group/Group.composition/Individ
ual/P.F/Rank/Sex/Size, family = gaussian(identity), data = Dataset)

Deviance Residuals:
    Min       1Q   Median       3Q      Max
-12.669   -3.727   -1.330    1.987   22.372

Coefficients:
                                                                                 Estimate  Std. Error t value Pr(>|t|)
(Intercept                                                                       3.095e+00  1.229e+00   2.518  0.012 *
Approaches                                                                       4.404e-01  1.855e-01   2.374  0.019 *
Proximity                                                                        2.134e-02  3.837e-02   0.556  0.57902
Approaches:Proximity                                                            -1.166e-03  3.948e-03  -0.295  0.76820
Approaches:Proximity:Aggression                                                  1.359e-03  9.707e-04   1.400  0.16393
Approaches:Proximity:Aggression:Group                                           -7.727e-04  5.077e-04  -1.522  0.13040
Approaches:Proximity:Aggression:Group:Group.composition                          1.543e-04  1.319e-04   1.170  0.24412
Approaches:Proximity:Aggression:Group:Group.composition:Individual               -2.222e-07  1.163e-06 -0.191  0.84882
Approaches:Proximity:Aggression:Group:Group.composition:Individual:P.F            5.073e-07  5.879e-07  0.863  0.38969
Approaches:Proximity:Aggression:Group:Group.composition:Individual:P.F:Rang       6.583e-10  3.383e-08  0.019  0.98450
Approaches:Proximit<:Aggression:Group:Group.composition:Individual:P.F:Rang:Sex  -2.271e-08  1.703e-08 -1.334  0.18455
Approach:Proximitiy:Aggression:Group:Group.composition:Individual:P.F:Rang:Sex:Size8.415e-10 3.218e-10  2.616  0.009**
```

```
---
Signif. codes:  0 '***' 0.001 '**' 0.01 '*' 0.05 '.' 0.1 ' ' 1

(Dispersion parameter for gaussian family taken to be 34.95904)

    Null deviance: 6579.0  on 144  degrees of freedom
Residual deviance: 4649.6  on 133  degrees of freedom
AIC: 940.32
```

Reduced GLMM with lowest AIC

```
Call:

glm(formula = Grooming frequency ~ Approach frequency * Proximity frequency/Group.composition/P.F/Sex/Size,
    family = gaussian(identity), data = Dataset)

Deviance Residuals:
     Min        1Q    Median        3Q       Max
-10.3949   -3.7105   -0.9086    2.2012   19.2137

Coefficients:
                                                  Estimate Std. Error t value Pr(>|t|)
(Intercept)                                      1.8905318  1.1860616   1.594 0.113250
Approaches                                       0.8174388  0.2084196   3.922 0.000138 ***
Proximity                                        0.0424714  0.0368332   1.153 0.250887
Approaches:Proximity                             0.0071064  0.0036463   1.949 0.053346 .
Approaches:Proximity:Group.composition          -0.0070900  0.0046142  -1.537 0.126710
Approaches:Proximity:Group.composition:P.F      -0.0011052  0.0030664  -0.360 0.719080
Approaches:Proximity:Group.composition:P.F:Sex  -0.0035040  0.0012978  -2.700 0.007809 **
Approaches:Proximity:Group.composition:P.F:Sex:Size  0.0002409  0.0000566   4.255 3.85e-05 ***
---
Signif. codes:  0 '***' 0.001 '**' 0.01 '*' 0.05 '.' 0.1 ' ' 1

(Dispersion parameter for gaussian family taken to be 31.76881)

    Null deviance: 6579.0  on 144  degrees of freedom
Residual deviance: 4352.3  on 137  degrees of freedom
AIC: 922.74
```

Complete GLM

```
Call:
glm(formula = Size ~ Approach frequency  + Grooming frequency +
    Proximity frequency, family = gaussian(identity), data = Dataset)

Deviance Residuals:
     Min        1Q    Median        3Q       Max
-14.2527   -4.0024    0.1624    5.4185    9.0032

Coefficients:
                      Estimate Std. Error t value Pr(>|t|)
(Intercept)           22.03370    0.98176  22.443  < 2e-16 ***
Approaches            -0.33806    0.07523  -4.494 1.45e-05 ***
Grooming               0.17582    0.08342   2.108   0.0368 *
Proximity             -0.15052    0.02580  -5.834 3.55e-08 ***
---
Signif. codes:  0 '***' 0.001 '**' 0.01 '*' 0.05 '.' 0.1 ' ' 1

(Dispersion parameter for gaussian family taken to be 35.99604)

    Null deviance: 7170.7  on 144  degrees of freedom
Residual deviance: 5075.4  on 141  degrees of freedom
AIC: 937.03

Number of Fisher Scoring iterations: 2
```
